# Antimicrobial Effect of Zn^2+^ Ions Governs the Microbial Quality of Donor Human Milk

**DOI:** 10.3390/foods10030637

**Published:** 2021-03-17

**Authors:** Carmel Hutchings, Zafnat Prokocimer Yair, Ram Reifen, Moshe Shemesh

**Affiliations:** 1 Department of Food Sciences, Institute for Postharvest Technology and Food Sciences, Agricultural Research Organization (ARO), Volcani Center, Rishon LeZion 7528809, Israel; carmel100@gmail.com; 2 School of Nutrition Science, Institute of Biochemistry, Food Science and Nutrition, The Robert H. Smith Faculty of Agriculture, Food and Environment, Institute of Biochemistry, Food Science and Nutrition, The Hebrew University of Jerusalem, Rehovot 761001, Israel; 3 Schneider Children’s Medical Center Israel, Emergency Medicine Department, Petah-Tikva 49202, Israel; zafnap@gmail.com

**Keywords:** human milk microbiota, human milk bank, *Bacillus cereus*, *Staphylococcus epidermidis*, antimicrobial minerals

## Abstract

Donor human milk (HM) obtained at HM banks is exceptionally crucial for the feeding and treatment of preterm infants. Bacterial contaminations of HM in various stages of its handling are very common and can lead to disqualification of donations or severe infections in worse cases. Hence, HM donations are subject to strict bacteriological evaluations pre- and post-pasteurization. The main contaminating species vary between countries, banks and donors and even exhibit inter-individual variation. We initiated an assessment of the bacteriological composition of HM donated by women hospitalized in a neonatal intensive care unit in Israel. The most common bacterium identified was *Staphylococcus epidermidis*, found in all but one of the HM samples; the presence of several species of coagulase-negative *Staphylococci* was also noted. Next, we sought to develop a platform towards antibacterial treatment using Zn^2+^ ions that have recently been found to be potent against contaminants isolated from bovine milk. Zn^2+^ efficiently inhibited the growth of viable aerobic population and *S. epidermidis* in HM. Growth was also inhibited in other Gram-positive bacteria such as *Bacillus cereus*, a well-known food-borne pathogen. *S. epidermidis* and *B. cereus* cells grown in the presence of zinc were taken for microscopic evaluation, aiming to demonstrate zinc’s antimicrobial mode of action morphologically. Images obtained using scanning electron microscopy indicated leakage of cellular content and cell lysis in *S. epidermidis*. Besides, *B. cereus* cells showed abnormalities in their cell surface and complete loss of flagella upon treatment with zinc. Along with the above findings, it should be noted that this was a pilot study that tested how high doses of Zn^2+^ affect breast milk as a product. Further research is likely needed on the safety of consumption of Zn^2+^-treated HM in infants and older children.

## 1. Introduction

Exclusive human milk (HM) feeding for the first six months of life, with continued breastfeeding for one to two years or longer, is considered as the recommended standard for infant feeding [1]. HM offers health advantages for all new-born infants but is particularly important for high-risk infants, especially preterm infants born at a very low birth weight (VLBW, <1500 g) [2]. The most recent policy statement from the American Academy of Pediatrics includes an explicit recommendation that all preterm infants should receive HM, with pasteurized donor HM being the preferred alternative in case the mother is unable to provide an adequate volume of milk [3]. The most vital benefits of this practice include decreased rates of late-onset sepsis and necrotizing enterocolitis (NEC) [4,5]. To offer these opportunities to preterm infants, HM should be obtained from milk banks. HM banks act to recruit and screen donors, collect and treat the milk donations (including bacteriologic screening, pasteurization and storage) and finally, distribute the donated milk to the appropriate treatment units [6].

Being a nutrient-rich biological product, HM is highly prone to bacterial contaminations. Exposure to high bacterial load can put preterm infants at risk, having an immature immune system. Therefore, once admitted, each donation goes through a strict bacteriological screening, and any donation with a bacterial load above the limit is discarded [7,8]. Guidelines published by the Israeli Ministry of Health for the forthcoming establishment of the first human milk bank in Israel state that bacteriological screening should test for total aerobic colony count, *Staphylococcus aureus* and *Enterobacteriaceae* before heat treatment (Table 1) [9]. Bacteriological testing should be performed after pasteurization as well, with any sample showing bacterial presence being discarded. Human milk banks in different areas worldwide are regulated differently, and guidelines for microbial limits and screening methods vary accordingly [10]. Pre- and post-pasteurization screenings have been shown to result in up to approximately 30% of donated milk being discarded [11,12]. Bacterial strains found in bacteriological samplings and contamination sources in HM banks are diverse, although typically, donor HM is colonized with normal skin flora before pasteurization [6]. However, variation between countries, banks and donors and even inter-individual variation are common, in regards to the abundance of different bacterial species in HM [12,13]. The most common pasteurization practice in treating HM is “Holder” pasteurization, involving heat treatment of 62.5 °C for 30 min [8]. This method has been shown to eliminate most pathogenic bacteria found in breast milk, yet it does not destroy sporulating bacteria, such as *Bacillus cereus* [14,15]. As a result, *B. cereus* spores in raw milk are a major source for its later presence in pasteurized HM [16].

The use of mineral and metal compounds as antimicrobial agents has been recognized for a long time, only to be replaced by organic antibiotics in the mid-20th century [17]. It is now common knowledge that a variety of metal ions are toxic to bacteria [18,19]. The effect of Mg^2+^ ions was recently investigated, and they were found to notably mitigate biofilm formation by *Bacillus* species [20,21]. Furthermore, it has been reported lately that Zn^2+^, another bi-valent cation, can efficiently mitigate biofilm formation and reduce heat resistance in *Bacillus* species [22]. Regardless of their crucial role in many cellular processes, Zn^2+^ ions may harbor some cellular toxicity at certain doses. It appears that the mode of antimicrobial action of Zn^2+^ ions is related to various cellular targets harboring multiple reactions and responses [23,24]. Besides, it should be noted that Zn^2+^ ions might also inhibit some of the non-harmful bacteria that are present in HM since these ions, apparently, possess a wide-spectrum of antimicrobial activity. 

As an extension to our previous work [22], we further assessed the antimicrobial ability of Zn^2+^ ions in HM. Since zinc has been proven to be potent against bacterial species isolated from bovine milk, we sought to test its activity against contaminating bacteria originating from HM, thereby elaborating our understanding regarding its mode of antimicrobial action. Thus, we aimed to identify major contaminants of HM to evaluate zinc’s antimicrobial effect against them. It is conceivable that the findings of this study will pave the way for the possible application of the antimicrobial activity of Zn^2+^ ions to improve the microbiological quality of HM in conjunction with classical pasteurization methods.

## 2. Materials and Methods 

### 2.1. Isolation and Identification of Bacteria from HM

HM samples were collected from women hospitalized at the Beilinson Women Hospital in Petach Tikva, Israel. Fourteen randomly selected raw HM samples were defrosted after being stored at temperature of −80 °C. Aliquots of 100 µL were plated on four types of agar plates: Lysogeny broth (LB, Difco, Le Pont de Claix, France), PCA (Oxoid, Basingstoke, UK), brain heart infusion (BHI) and De Man, Rogosa and Sharpe (MRS, Himedia, Mumbai, India), then incubated at 37 °C for 48 h. Based on differentiating morphologies, colonies were selected from each agar plate. The selected colonies were then streaked and grown separately on agar plates to assure purity. Following separate growing, colonies were identified using matrix-assisted laser desorption/ionization MALDI TOF mass spectrometry (Bruker Daltonik GmbH, Bremen, Germany). Isolates were prepared for the analysis as follows: the selected colonies were suspended in 300 µL pure - polymerase chain reaction (PCR)-grade water in 2 mL sterile plastic tubes, along with 900 µL of ethanol. Tubes were then centrifuged at 13,000× *g* for 2 min, the supernatant was discarded and the tubes were left to dry in a biological safety cabinet. Next, 10 µL of 70% formic acid was added to the tubes and briefly mixed, 10 µL of pure acetonitrile was added and the tubes were mixed again. The tubes were centrifuged at 13,000× *g* for 2 min; 1 µL from the supernatant was loaded into the MALDI Biotyper reading plate, dried and then covered with 1 µL of HCCA solution. Plate reading was done using a Bruker Daltonik MALDI Biotyper and analyzed with the following MSP libraries: BDAL, mycobacteria library (bead method), filamentous fungi and listeria. The results were then transferred to a Krona visualization program. Strains identified by the instrument as “highly probable species identification” (score value > 2.3, scale of 0–3.0) were included and listed for their presence in the original sample. Isolates were taken for further identification using 16S rRNA sequencing in order to assure that the identification was accurate.

### 2.2. Growth Medium and Conditions

Bacterial strains were propagated in LB consisting of 10 g of tryptone, 5 g of yeast extract and 5 g of NaCl per liter, or on solid LB medium supplemented with 1.5% agar. Each bacterial strain was plated on the solidified LB plates at 37 °C until visible growth of bacterial colonies occurred. Afterwards, a fresh starter culture was generated from a single bacterial colony, by incubating it in LB broth overnight at 23 °C with shaking at 150 rpm. Being a highly soluble compound and an FDA generally recognized as safe (GRAS) approved for food, we chose to use ZnCl_2_ as the source for Zn^2+^ ions. Addition of Zn^2+^ ions was performed by diluting a 1 M solution of ZnCl_2_ (Sigma-Aldrich, St. Louis, MI, USA) directly into the growth medium to achieve a desirable experimental concentration. For evaluation of bacterial growth in isolates in HM, milk was first heat-treated in order to eliminate any previous bacterial presence. Pasteurization of HM was done using “Holder” pasteurization, i.e., heat treatment at 62.5 °C for 30 min.

### 2.3. Growth Analysis of General Viable Bacterial Population in HM

Three different HM samples were pooled into a sterile container, then divided into three Erlenmeyer flasks, each flask containing 5 mL of HM. Zn^2+^ was added to separate flasks at concentrations of 1, 3 and 5 mM from 1 M stock of ZnCl_2_; an additional unsupplemented HM flask was used as control. Concentrations were selected based on previous work [22], adjusting them as efficient against most contaminating species. The flasks were then incubated at 37 °C with shaking at 150 rpm. Every 2 h, 100 µL of each sample was taken for quantification of bacteria, and viable cells were determined by the CFU counting method similar to that as previously reported [22].

### 2.4. Growth Analysis for HM Isolates 

Two major bacterial species, *B. cereus* and *Staphylococcus epidermidis*, isolated from HM and identified using a MALDI TOF biotyper, were grown in a LB medium. For starter cultures, strains were grown as described above, then introduced into the LB medium in 1:100 ratio, with or without supplementation of zinc. ZnCl_2_ was added at concentrations of 1, 3 and 5 mM; flasks were then incubated at 37 °C with shaking at 150 rpm. 100 µL of each sample was collected every 2 h for the duration of 8 h. Viable cell count was determined using the CFU method as previously described. For evaluation of bacterial growth of the isolates in HM, a similar assay was conducted using pasteurized HM, with pasteurization being done as described above. 

### 2.5. Scanning Electron Microscopy (SEM) Analysis for Visualization the Morphological Changes of Bacterial Cells 

The bacterial cells were prepared and visualized using the SEM observation similar to that as reported recently [22]. 

### 2.6. Statistical Analysis

The experimental data were analyzed through ANOVA following post hoc *t*-tests using Microsoft Excel 2010 and GraphPad Prism 6 software. *p*-Values < 0.05 were considered significant. The results are based on three biological repeats performed in duplicates.

## 3. Results

### 3.1. Isolation and Identification of Bacterial Species Contaminating in HM

**Figure 1:**
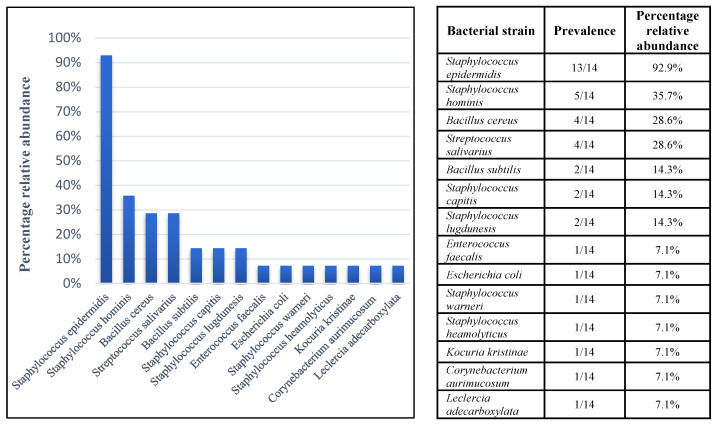
Bacterial strains identified in pumped human milk. Percentage relative abundance represents proportion of samples in which bacterial strain was identified out of overall samples tested (*n* = 14). Identification of strains was initially done using the MALDI-TOF Scheme and then through 16S rRNA sequencing analysis. Bacterial strains mentioned were identified with score values > 2.3.

As previously mentioned, HM stored in HM banks is highly prone to contaminations arising from its biologically rich composition. At the same time, the core contaminating bacterial strains may vary between different banks and countries of origin [25,26,27]. Therefore, it was decided to initiate a local sampling of donated HM from women hospitalized in the same unit in order to gain better insight into local contaminants, while also aiming to acquire isolates originating from HM. To do so, 14 randomly selected samples of HM donated from different women were plated on agar plates of various growth mediums, incubated for 48 h, then identified using a MALDI TOF biotyper. Figure 1 shows bacterial strains found in the tested samples and identified as “highly probable species identification” (score value > 2.3), presented by relative abundance. Overall, 13 strains were identified; *S. epidermidis* was the bacterial strain with the highest prevalence as it was present in 13 out of the 14 milk donations sampled. 16S rRNA sequencing results matched those received from the MALDI TOF assay. The prevalence of bacterial species among our samples was similar to those previously reported; the majority of bacterial strains identified belonged to the *Staphylococcus* genus and seemingly originated from commensal skin flora [28,29,30]. *S. epidermidis* is a non-motile, Gram-positive, commensal microorganism that often colonizes the skin and mucous membranes of mammals and is the most prevalent staphylococcal species found in humans [31]. Although its presence is considered to be harmless and even, in some cases, beneficial to human health, *S. epidermidis* has been identified as the most common cause for primary bacteremia and infection of indwelling medical devices, particularly in immunocompromised individuals and neonates [32]. Moreover, *S. epidermidis* isolates from HM originating from healthy women have shown resistance to various types of antibiotics [33]. Another major issue in the management of milk banks revolves around the presence of sporulating bacterium, *B. cereus*, in pasteurized HM. Therefore, isolation and identification by sequencing was also done for *B. cereus* isolates, intending to use them to test zinc’s effect against this bacterium. Hence, the ability of Zn^2+^ to mitigate microbial contaminations in HM was further tested against *S. epidermidis* and *B. cereus.*

### 3.2. Zn^2+^ Ions Inhibit Bacterial Growth in HM

**Figure 2:**
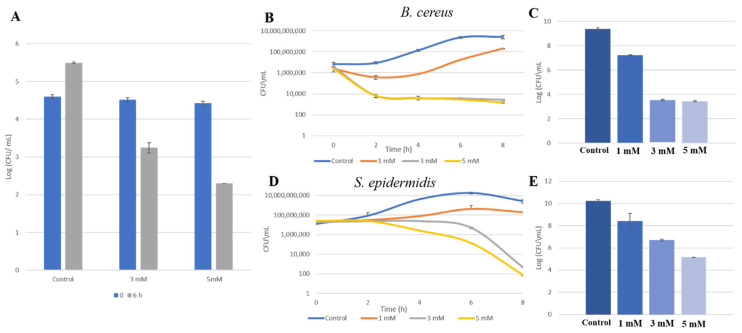
Antimicrobial effect Zn^2+^ ions on bacterial contaminants in human milk (HM). The effect of Zn^2+^ ions on growth of aerobic bacterial population in HM; growth was measured after 6 h of incubation of raw HM, with or without the addition of zinc (**A**). Growth curve analysis of *B. cereus* (**B**) and *S. epidermidis* (**D**) cells isolated from HM grown for 8 h with various concentrations of zinc in Lysogeny broth (LB) medium. Bacterial levels of *B. cereus* (**C**) and *S. epidermidis* (**E**) grown with or without zinc in pasteurized HM after 8 h. *p*-value < 0.05, compared with control. Error bars represent standard deviation (SD). CFU = colony-forming units.

First, to evaluate the antimicrobial activity of zinc in HM, we wanted to initially test the effect of Zn^2+^ ions on the growth of general viable bacterial populations in pooled unpasteurized HM. Following that, we tested zinc’s activity against *S. epidermidis* and *B. cereus* previously isolated from HM, first in growth medium, then in pasteurized HM. As can be seen in Figure 2A, addition of zinc to unpasteurized HM in concentrations of 3 mM and 5 mM efficiently reduced bacterial levels of general viable bacterial populations, and growth was noticeably withheld after 6 h of incubation with zinc. Notably, the effect of 1 mM of zinc is not shown in this analysis since it was found to be similar to that of 3 mM. Next, a growth-curve analysis of *B. cereus* and *S. epidermidis* was done by growing the isolates in a growth medium for 8 h, with or without the presence of zinc. An examination of zinc’s effect on their growth in HM medium was done next. As can be seen in Figure 2B, bacterial cells of *B. cereus* were able to grow in the presence of 1 mM of zinc, although to a lesser extent compared to the control, and it seemed that growth was somewhat delayed in the overall period of incubation. On the other hand, growth was completely inhibited when *B. cereus* cells were exposed to 3 and 5 mM of zinc. Figure 2C shows bacterial levels after 6 h in which bacterial cells were grown in pasteurized HM, showing a 2-log reduction when cells were exposed to 1 mM of zinc and a massive 6-log reduction in 3 and 5 mM of zinc. A similar trend was evident when *S. epidermidis* cells were grown in the presence of zinc, with a concentration-dependent reduction in bacterial levels (Figure 2D,E). However, compared to *B. cereus* cells, it seems that a longer incubation period was required for zinc to exert its antimicrobial effect against this strain, as a reduction in bacterial levels started only after 6 h and continued to decline for the rest of the incubation period. Overall, there was a prominent inhibition of bacterial growth in all of the conduct assays, regardless of bacterial strain and growth medium.

### 3.3. Zn^2+^ Ions Cause Morphological Deformation in B. cereus and Cell Lysis in S. epidermidis 

**Figure 3:**
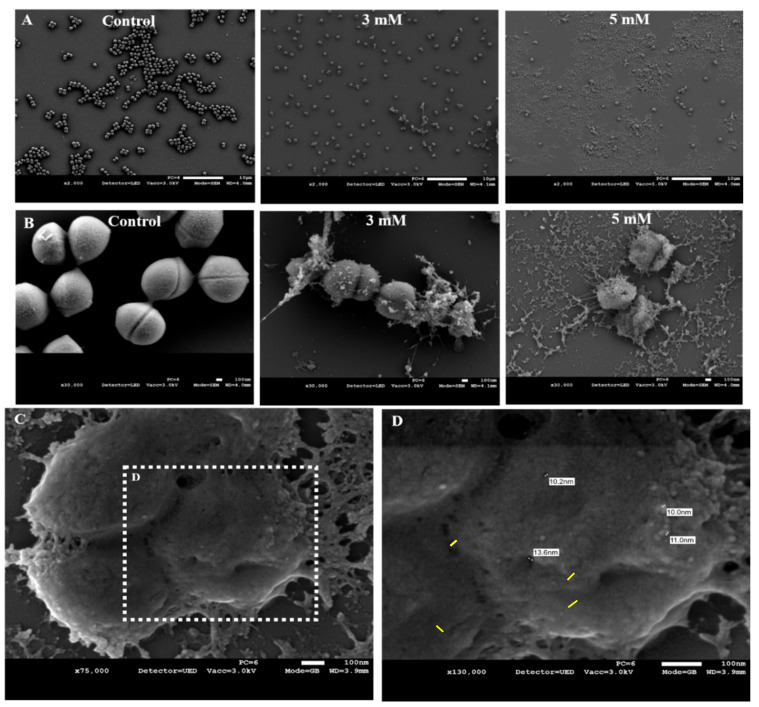
Zn^2+^ ions cause lysis of *S. epidermidis* cells. SEM images of *S. epidermidis* cells following overnight exposure to ZnCl_2_. Images shown were taken at magnifications of 2000× (**A**), 30,000× (**B**) with a Jeol JSM-IT-100 LV at 5.0–10.0 kV. Magnification at 75,000× (**C**) and 130,000× (**D**) of cells grown with 5 mM of ZnCl_2_. Yellow lines indicate size of cavities (left) and approximate size of leaked content (right).

Microscopic evaluation can reveal morphological changes caused by exposure to zinc and improve our understanding of the molecular basis of its antimicrobial effect. Our previous work showed that *Bacillus subtilis* cells grown with various concentrations of zinc were significantly shorter than bacterial cells grown in its absence [22]. Imaging with electron microscopy showed that this was not the case with the HM isolates. Both strains were grown overnight with 3 and 5 mM of zinc, then taken to scanning electron microscopy (SEM) visualization. SEM imaging of *S. epidermidis* revealed a dramatic effect caused by the presence of zinc, as cells treated with 3 and 5 mM displayed loss of cellular shape and integrity and leakage of cellular content, resulting in a complete lysis of cells (Figure 3A,B). A closer inspection of the damaged cells showed that exposure to Zn^2+^ ions has seemingly caused the formation of numerous small cavities over the cells surface, possibly promoting the noted leakage of cellular content (Figure 3C,D). The leaked content found spread over the cell surface was measured at approximately 10 µm, similar to the size of the the formed cavities (Figure 3D).

**Figure 4:**
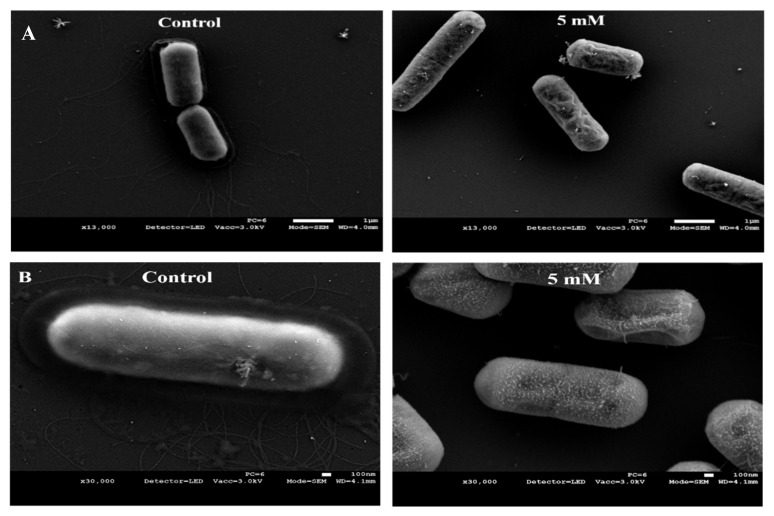
Zn^2+^ ions affect cell surface structure and generation of flagella in *B. cereus* cells. SEM images of *B. cereus* cells following overnight exposure to 5 mM of ZnCl_2_ showing abnormalities in cell surface, as well as complete loss of flagella. Images shown were taken at magnifications of 13,000× (**A**) and 30,000× (**B**) with a Jeol JSM-IT-100 LV at 5.0–10.0 kV.

Imaging of *B. cereus* cells did not yield an effect as explicit as that seen with *S. epidermidis* cells, thus not demonstrating clear morphological changes and a possible mode of action. Also, distinctions between control and treated cells were only evident when cells were exposed to 5 mM ZnCl_2_. The first apparent difference between bacterial cells in the control and cells grown with zinc was the integrity of the cell surface (Figure 4). Cell surface in the zinc-treated bacteria was considerably more abnormally textured, as opposed to the smooth cell surface seen in the control. The other obvious difference between the control and the zinc-grown bacteria was evident loss of flagella. Cells in the control were able to form numerous long and branching flagella. On the other hand, no flagella were found emerging from bacteria cells exposed zinc. Comparing images of *S. epidermidis* cells to *B. cereus* cells exposed to Zn^2+^ ions illustrates a species-dependent impact. This further strengthens the notion that zinc’s antimicrobial activity involves various mechanism and cellular targets and emphasizes its complexity.

## 4. Discussion

For the most part, samples of donor HM prior to pasteurization are colonized with normal skin flora, including coagulase-negative *Staphylococcus* and *Streptococcus* species, while some Gram-negative bacilli are also common to find [6]. Normally, the HM microbiota is predominantly comprised of commensal bacteria such as coagulase-negative *Staphylococcus*, *Lactobacillus* spp. and *Bifidobacteria* spp., hence their presence in pumped HM is very likely. Previous studies also marked the presence of *Acinetobacter* spp., *Escherichia coli*, *Pseudomonas* spp., *Klebsiella* spp., *Enterococcus* spp., *Enterobacter* spp., *Bacillus* spp. and *Moraxella* spp. [25,34,35,36]. All are considered commensals yet can be pathogenic. Contamination of HM may occur during pumping, collection, transport or storage, and bacteria can originate from the mothers’ skin, breast-pump components or milk containers [28]. The first target of our study was to examine the microbial flora of expressed HM in our region, looking to mark the main contaminants. Indeed, the most common bacteria identified was *S. epidermidis*, found in all but one of the HM samples. In accordance with previous reports, coagulase-negative *Staphylococcus* spp. were also notably abundant in our sampling, and six species belonging to this genus were spotted. Our study was carried out using HM donated by women hospitalized in the neonatal intensive care unit (NICU). One previous study showed comparable results, as the second-most frequent bacteria isolated from the expressed HM was *S. epidermidis* [27]. On the other hand, the most abundant bacteria identified in the same study was *Klebsiella* spp., which was not found in any of the HM we sampled. This further stresses the diversity in bacterial populations that can be found in bacteriological screenings in expressed HM, which can be credited to various factors. *S. epidermidis* and other coagulase-negative *Staphylococcus* are considered commensal among most milk banks’ criteria, yet they may cause nosocomial infection in preterm infants. However, since they are most likely to be eliminated by pasteurization, the main concern is that their presence will lead to disqualification of donations. 

Post-pasteurization bacterial presence is not as common since most bacteria populating raw HM do not survive Holder pasteurization [6,8]. Still, some pasteurized milk samples do come out positive after testing for bacterial presence. In most cases, the predominant contaminants found after pasteurization belong to the *Bacillus* spp. Furthermore, in some cases, HM samples that showed no bacterial presence before pasteurization will test positive for *Bacillus* spp. after the heat treatment [36]. This can be attributed to the germination of dormant spores into vegetative cells following exposure to heat, which is common for this species [37]. In this context, a major concern in the management of HM banks will be the post-pasteurization presence of *B. cereus*. This bacterium is highly pathogenic in preterm infants; in 2013, two infants born at very low birth weight presented with severe intestinal infection after consuming donated HM contaminated with *B. cereus* in an NICU in France [38]. Retrospective analysis revealed that the HM batch contained *B. cereus* at levels below the detection threshold of the reference method. 

Crossing results of the HM bacteriological analysis with previous reports marked *S. epidermidis* and *B. cereus* as solid candidates to exemplify zinc’s effect against the major and common HM contaminants, both before and after pasteurization. As we expected, both strains were highly sensitive to zinc, as the presence of Zn^2+^ ions in various concentrations inhibited their ability to grow in a growth medium and in pasteurized HM. After observing the bactericidal effect caused by exposure to zinc, we wished to further elaborate its antimicrobial effect. A closer inspection of zinc’s influence on bacterial cells was achieved using scanning electron microscopy, which demonstrated a diverse strain-dependent antimicrobial effect. The antimicrobial effect of zinc over *S. epidermidis* has been previously described [39], while its requirement for proper cellular processes and defense mechanisms, such as biofilm formation, has also been reported [40,41]. Zinc is an essential element for all organisms that include microorganisms, as it is involved in many vital cellular reactions when found in low endogenous concentrations [42,43]. Zinc uptake by cells is regulated, but it does not affect its adsorption to cell membranes. The increase of Zn^2+^ concentrations above optimal levels disturbs this homeostasis and allows entry of Zn^2+^ ions to inside the cells, resulting in a cytotoxic effect. The antimicrobial mode of action for zinc against various bacterial and fungal species has been addressed before. It has been suggested that zinc’s antimicrobial activity can be a result of the multiple inhibitory effects this ion has on crucial metabolic processes in bacterial cells, such as glycolysis, glucosyltransferase production, polysaccharide synthesis, transmembrane proton translocation and acid tolerance [24]. Furthermore, Zn^2+^ ions can also enhance proton permeabilities of bacterial cells’ membranes and reduce adenosine triphosphate (ATP) synthesis in glycolyzing cells. Disturbance to energy-producing and respiratory processes in bacterial cells can be a result of zinc’s ability to inhibit the activity of key enzymes, such as glycolytic enzymes [23]. Another suggested mechanism is a direct interaction of the ions with microbial membranes, leading to an enhanced permeability and membrane destabilization [44,45]. In a study conducted using a Ag-ZnO nanocomposite intending to demonstrate the compound’s antimicrobial effect using *S. aureus* and *E. coli* cells, upon treatment, bacteria displayed strong evidence of membrane disorganization and increased roughness, leading to a leak-out of the intracellular components and subsequent cell lysis in both strains [45]. These authors reported that cell death was induced by either strong electrostatic interaction between the Zn^2+^ ions and the negatively charged cell membrane of the bacterial cells or via production of intracellular reactive oxygen species (ROS). Similar mechanisms of action can be advanced to explain the cell lysis of *S. epidermidis* observed in our study. 

Unlike *S. epidermidis* cells, *B. cereus* cells did not show a phenotype of cell lysis when grown in the presence of zinc, despite showing a bactericidal effect when treated with the same concentrations. Interestingly, when exposed to 5 mM of ZnCl_2_, cells showed abnormalities in the cell surface, as well as complete loss of flagella. Not much is known about the relation between ions’ homeostasis and flagella formation in bacterial cells. Zn^2+^ ions have been reported to be crucial for the activity of anti-sigma factors in various species [46,47]. In *B. cereus*, flagella formation is linked to the activity of Sigma 54, a sigma factor which is somewhat characterized as a modulator of bacterial cell exterior [48]. A phenotype similar to ours, showing extensive loss of flagella in *B. cereus* ATCC 14579, was obtained after deletion of the *rpoN* gene encoding for sigma 54 [49]. Alterations in intracellular zinc levels caused by over exposure to Zn^2+^ ions can possibly promote the expression of anti-sigma factors and subsequently, suppress the activity of sigma 54, causing for a reduction in flagella biosynthesis. However, a direct effect of Zn^2+^ ions on the levels of anti-sigma factors or on the expression of the *rpoN* gene in *B. cereus* has not been tested. The detailed mechanism by which Zn^2+^ ions affect physiological processes such as flagella formation in *B. cereus* needs further research as literature on this subject is lacking. Furthermore, the microscopically visible changes in bacterial cell morphology obtained after exposure to zinc does not necessarily account for the observed bactericidal effect. Hence, this promotes the notion that, in *B. cereus* cells, the antimicrobial effect of Zn^2+^ ions, resulting in cell death, could likely be related to some intracellular changes in physiological state of the cell.

It should be noted that the current investigation was designed as a proof-of-concept study, aiming to evaluate the feasibility of a zinc-based treatment in targeting bacterial species populating pumped HM. Thus, the results of this study open a possibility for developing a mineral-based antimicrobial approach that can possibly offer a future solution to microbiological problems in the food industry, including HM handling. Incorporation of minerals can be done in various stages of food preparation and treatment, as different stages can be the source for bacterial contaminations. Previously, it was reported that incubation with magnesium or zinc before heat treatment led to a reduction in post-pasteurization survival rates in *Bacillus* spp. [21,22]. Hence, it could be suggested that the supplementation of milk with Zn^2+^ ions before heat treatment should lead to a significant reduction in post-pasteurization survival rates of spoiling *Bacillus* species. Enrichment of HM with zinc could be fundamental for not only the bacterial safety of the milk, but also for the prevention of zinc deficiencies, especially considering the fragility of the target recipients. Zinc is a critical micronutrient for preterm infants, having a significant role in immune function and growth [50]. Moreover, zinc deficiencies, especially if occurring in the neonatal period, are often associated with predispositions to pathologies such as autoimmunity and autism [51,52]. Evidence suggests that the zinc levels in HM may not be optimal for preterm infants and that human-milk feedings should be fortified with additional zinc [53]. However, several issues need to be addressed regarding the application of zinc as an antimicrobial aimed at HM treatment. A practical review published by the world health organization (WHO) concerning optimal feeding of low-birth-weight infants acknowledged that it is standard practice in many neonatal units to give VLBW infants a multicomponent fortifier with HM, providing an additional 0.5–1.8 mg/kg/day of zinc, until the infant reaches a weight of 1800–2000 g [54]. Our study was conducted using a high-dose Zn^2+^ supplementation, reaching up to 5 mM (i.e., 327 mg/L). Such a dose is substantially higher than the physiologic requirement for growth, hence concerns about toxicity and other complications need to be evaluated. For instance, in adults, high doses of zinc caused inhibition of absorption of other micronutrients (such as Cu^2+^ and Fe^3+^), which could also be the case in preterm infants [55,56]. Therefore, doses of Zn^2+^ supplementation should be lowered upon optimization of possible treatment, along with ruling out any negative effects of this practice on infants’ health. Optimization and further research should also include use of other zinc compounds, namely those used as dietary supplements, which might exert similar antimicrobial ability with lower risks and costs. 

## Figures and Tables

**Table 1:** Israeli Ministry of Health guidelines for microbial limits of donor human milk, in milk bank operation [9].

Pre-Pasteurization	Post-Pasteurization
Total colony count < 10^5^ CFU/mL	Total colony count < 10 CFU/mL
*Enterobacteriaceae* < 10^4^ CFU/mL	
*Staphylococcus aureus* < 10^4^ CFU/mL	

CFU = colony-forming units.

## Data Availability

The datasets generated and analyzed during the current study are available from the corresponding authors on reasonable request.
